# JAK4D, a first-in-class thyrotropin-releasing hormone analogue, reverses scopolamine-induced memory deficits

**DOI:** 10.1093/braincomms/fcag006

**Published:** 2026-01-16

**Authors:** Roisin McMackin, Smita Price, Gillian R Slator, Orla Hardiman, Julie A Kelly

**Affiliations:** Academic Unit of Neurology, School of Medicine, Trinity Biomedical Sciences Institute, Trinity College Dublin, University of Dublin, Dublin 2 D02 R590, Ireland; Discipline of Physiology, School of Medicine, Trinity Biomedical Sciences Institute, Trinity College Dublin, University of Dublin, Dublin 2 D02 R590, Ireland; Smita Price Associates Ltd., Great Dunmow, Essex CM6 1JE, UK; Academic Unit of Neurology, School of Medicine, Trinity Biomedical Sciences Institute, Trinity College Dublin, University of Dublin, Dublin 2 D02 R590, Ireland; Academic Unit of Neurology, School of Medicine, Trinity Biomedical Sciences Institute, Trinity College Dublin, University of Dublin, Dublin 2 D02 R590, Ireland; Academic Unit of Neurology, School of Medicine, Trinity Biomedical Sciences Institute, Trinity College Dublin, University of Dublin, Dublin 2 D02 R590, Ireland; Neuropath Therapeutics Limited, Dublin 2 D02 X361, Ireland

**Keywords:** thyrotropin releasing hormone, JAK4D, pro-cognitive effects, scopolamine-challenge, neurodegenerative disease

## Abstract

There is a pressing unmet clinical and health economic need for effective drugs to treat cognitive impairment that occurs in neurodegenerative diseases. JAK4D is a first-in-class thyrotropin releasing hormone (TRH) analogue that overcomes the pharmacological limitations of thyrotropin releasing hormone and enables delivery of the long-recognized multifactorial neurotherapeutic actions of thyrotropin releasing hormone without inducing endocrine side effects. JAK4D is demonstrated to be neuroprotective and significantly reduce excitotoxic-induced hippocampal-dependent memory deficits in rat. In the present study, we used the scopolamine challenge test coupled with the novel object recognition test to evaluate the effect of JAK4D on scopolamine-induced recognition memory deficits in the male, Lister-Hooded rat. Scopolamine administration has been shown by others to mimic cholinergic and brain network disruption in neurodegenerative diseases. Although the scopolamine challenge test does not fully replicate the pathophysiology of neurodegenerative disease, such as Alzheimer’s disease, it is a well-recognized acute pharmacological model for assessing the ability of pharmacological interventions to counteract memory deficits relevant to neurodegenerative diseases. In this model of cholinergic dysfunction, we also assessed the effects of thyrotropin releasing hormone, taltirelin (a degradation-stabilized thyrotropin releasing hormone analogue) and the acetylcholinesterase inhibitor, donepezil, as a positive reference compound. The discrimination (d2) index was used as the primary measure to assess the effect of treatment on scopolamine-induced performance deficit in the novel object recognition test. d2 is a standard well-recognized measure of discrimination between a novel and familiar object in the novel object recognition test, which advantageously takes into account individual differences in exploration levels. Across all investigations, JAK4D (1 mg/kg i.p.) significantly reversed scopolamine-induced recognition memory impairment (*P* = 0.0274, *P* = 0.0002, *P* < 0.0001). The degree of reversal of scopolamine-induced memory deficits by JAK4D (1 mg/kg i.p.) was indistinguishable from that observed for donepezil (0.1 mg/kg p.o.) (*P* = 0.026). Subcutaneously administered JAK4D (0.3–10.0 mg/kg) also significantly reversed this deficit (*P* = 0.0432–0.0021). Furthermore, similar pro-cognitive effects were exerted by thyrotropin releasing hormone (5 mg/kg i.p., *P* = 0.0055) and taltirelin (10 mg/kg p.o., *P* = 0.0002). Together, these results underscore the relevance of the central thyrotropin releasing hormone signalling system for the treatment of memory impairment. Data from the current study provide further evidence in support of the potential of JAK4D as a novel therapeutic for cognitive deficits in neurodegenerative diseases.

## Teaser

McMackin *et al*. report that JAK4D, a first-in-class thyrotropin releasing hormone analogue, reverses scopolamine-induced memory impairment in the rat novel object recognition test, pointing to a novel therapy for cognitive deficits in neurodegenerative diseases

## Graphical Abstract

**Graphical Abstract:**
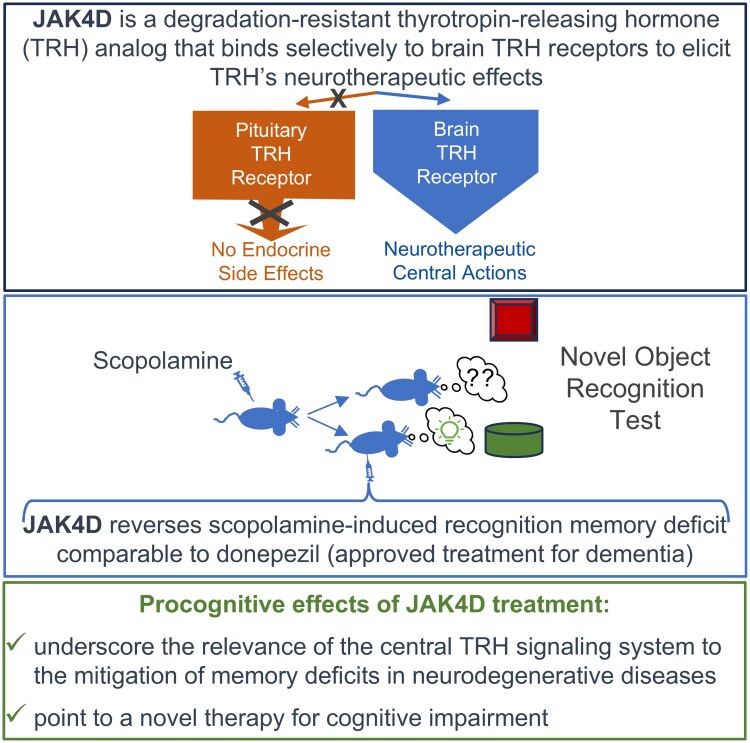

## Introduction

Cognitive impairment is a characteristic of many neurodegenerative diseases. It is perhaps most commonly associated with Alzheimer’s disease^1-3^ but also occurs in other neurodegenerative diseases, such as amyotrophic lateral sclerosis (ALS)^4-6^ and Parkinson’s disease.^7,8^ It may also arise as a consequence of traumatic brain injury.^9,10^ Cognitive deficits have a profound adverse impact on quality of life and caregiver burden,^11-13^ and there remains a pressing unmet clinical and health economic need for neurotherapeutic drugs that prevent or delay onset, relieve symptoms and/or slow loss of cognitive functioning.

The scopolamine challenge test is extensively used in the assessment of new prospective pro-cognitive drugs that may be effective in treating cognitive impairment. In this pharmacological model, administration of the muscarinic acetylcholine receptor antagonist scopolamine disrupts cholinergic signalling and temporarily impairs several cognitive domains relating to memory and executive function in the rat and in humans^14-17^. Reduced cholinergic signalling is a well-established driver of memory decline in Alzheimer’s disease^18-20^ and Parkinson’s disease^7,18,19^ and is also implicated in cognitive impairment following traumatic brain injury.^21^ In humans, intracranial brain recordings show scopolamine-induced memory impairment is associated with decreases in connectivity across the hippocampal formation.^22^ Moreover, magnetoencephalographic data from humans demonstrate that scopolamine produces temporary abnormalities in frontoparietal and temporal network activity that resemble those reported in Alzheimer’s disease patients, thus supporting the scopolamine challenge test as a psychopharmacological model of Alzheimer’s disease.^23^ Scopolamine-challenged rats also demonstrate patterns of cortical network dysfunction observed in Alzheimer’s disease.^23^ Although this acute behavioural pharmacological model of cholinergic dysfunction does not fully replicate the pathophysiology of chronic neurodegenerative diseases, such as Alzheimer’s disease, it simulates clinically relevant changes not only in cholinergic signalling but also in cognitive functioning and neuronal networking.

Notably, the work of others demonstrates that the patterns of parietooccipital network dysfunction induced by scopolamine^23^ also mimic those observed in ALS^24-29^ which is now known to be associated with cognitive and behavioural changes in approximately 50% of those affected,^6^ underpinned by such non-motor cortical network dysfunction.^24,30,31^ This aspect of the disease, however, has largely been disregarded in the development and testing of potential ALS therapies.^4,5^ Since the scopolamine challenge test mimics both ALS-related cortical network dysfunction and ALS-related cognitive symptoms, this model may also provide insights into the ability of drug treatment to have a beneficial impact on cognitive impairment, when present, in ALS patients.

We are investigating the central thyrotropin-releasing hormone (TRH) signalling system as an innovative mechanism for the treatment of cognitive impairment in neurodegenerative diseases. TRH, initially identified as a hypothalamic regulatory hormone controlling the release of thyrotropin stimulating hormone (TSH) from the pituitary, is also a centrally acting neuropeptide with multifactorial neurotherapeutic, homeostatic, neuromodulatory and neuroprotective effects in extrahypothalamic brain areas.^32-34^ Activation of the central signalling system of TRH counteracts numerous Alzheimer’s disease-related pathophysiological mechanisms.^35-39^ Moreover, TRH exerts stimulant action on cortical and hippocampal cholinergic pathways,^40^ increases acetylcholine production,^41^ potentiates the excitatory actions of acetylcholine on cortical neurons,^42^ improves cerebral blood flow and bioenergetics,^43^ promotes arousal and attention through modulation of histaminergic and orexin neurons^44-46^ and elicits ergotropic actions in the CNS.^47^ TRH has also been shown to decrease glutamate release while increasing the release of GABA, potentially mitigating disinhibition and excess excitatory signalling, which occurs in both Alzheimer’s disease^48^ and ALS.^49^ Despite TRH inherently being a poor drug, treatment with native TRH attenuates memory impairment in healthy volunteers treated with scopolamine,^50^ and also elicits a significant increase in arousal and affect and improvement in semantic memory in people with Alzheimer’s disease.^51^

As observed for other therapeutic neuropeptides, such as glucagon-like peptide-1 (GLP-1), the clinical use of native TRH presents pharmacological challenges, due in part to its rapid degradation by TRH-degrading ectoenzyme (TRH-DE) (EC 3.4.19.6) and its serum counterpart, thyroliberinase.^52-54^ Additionally, native TRH evokes undesirable endocrine actions via pituitary TRH receptors. These endocrine effects critically limit clinical exploitation of the neurotherapeutic potential of TRH, such that extensive efforts were made across the 1980s and 1990s to develop degradation-stabilized TRH analogues that could evoke central TRH neurotherapeutic actions without stimulating endocrine effects.^37,55,56^ Taltirelin (TA-0910, marketed as Ceredist), the only TRH analogue to have reached the market, was launched exclusively in Japan for the treatment of spinocerebellar ataxia in 2000.^33,57^ Taltirelin, however, retains the ability to bind pituitary TRH receptors and stimulate TSH release, though with weaker activity than TRH.^58-60^

More recently, a series of first-in-class TRH-based compounds have been developed that overcome the obstacles of enzymatic degradation and endocrine side effects.^55,61^ These compounds uniquely incorporate a dual function pharmacophore for potent inhibition of TRH-DE, and nanomolar affinity (K_D_ 7 nm) and selectivity for central TRH receptors in rat and human brain.^55,61^ Importantly, these compounds do not signal through pituitary TRH receptors that mediate TRH’s endocrine effects. Our previous studies demonstrate that the lead compound of this series, JAK4D (Glp-Asn-Pro-D-Tyr-D-TrpNH_2_), evokes multiple neurotherapeutic effects across a number of rodent models of neurodegeneration.^55^ For example, in a kainate-induced model of oxidative stress and neurodegeneration, JAK4D significantly reduced hippocampal-dependent cognitive deficits, free radical release and neuronal cell loss. Additionally, JAK4D reduces motor decline, weight loss and lumbar spinal cord neuronal loss in the G93A-SOD1 mouse model of ALS.^55^ The reduction of neuronal loss and mitigation of pathophysiological processes leading to cell death observed in these prior studies indicate that JAK4D may potentially present disease modifying effects, in addition to symptomatic relief.

The current studies were undertaken to determine if activation of the central TRH signalling system by JAK4D reverses scopolamine-induced memory impairments, as measured by the novel object recognition (NOR) test.^62,63^ The NOR test is a well-characterized, method for evaluating non-spatial visual memory that relies on rodents’ natural proclivity for exploring novelty, and is widely used for evaluating drug effects on memory and recognition (as reviewed in^62-64^). This test closely resembles those used in studying human cognition, increasing the ecological validity of the test, compared to many other rodent memory tests.^65^. Furthermore, administration of scopolamine is demonstrated to impair performance in this test.^62,66^

In addition to evaluating the effects of JAK4D on scopolamine-induced recognition memory deficits in rats, we also assessed the effect of TRH, taltirelin and, as a benchmark, donepezil (Aricept)—a reversible acetylcholinesterase inhibitor approved for treatment of dementia in mild, moderate and severe Alzheimer’s disease.

## Materials and methods

All experiments were conducted by Transpharmation Ltd. (The London Bioscience Innovation Centre, London NW1 0NH, United Kingdom), employing standard protocols, in accordance with the U.K. Animals (Scientific Procedures) Act 1986 and European Directive 2010/63/EU, UK Home Office Guidelines and Codes of Conduct and in compliance with the ARRIVE guidelines.

### Animals

Male, Lister-Hooded rats (200–220 g at time of delivery) naïve to drug treatment from Envigo UK were used. According to U.K. Royal Veterinary College (RVC) guidelines, animals were allowed to acclimatize for 7 days. Animals were housed with bedding and nesting from arrival and throughout the study. Rats were housed with no environmental enrichment in open top conventional cages (51 d × 22 w × 22 h cm) in groups of four with food (5CR4—Certified CR 14% Protein Rodent Diet from LabDiet) and water available ad libitum. As approved by UK Home Office Project License held by Transpharmation, the animals did not have any enhanced enrichment, such as tubes and chewsticks etc., as this has a negative impact on the test where animals are required to show good exploratory behaviour in a novel environment. Animals were on a 12 h/12 h light dark cycle daily with light on at 7 am and off at 7 pm. All assays were run between 9 am and 4 pm.

### Chemicals, drug preparation and administration

**Figure 1:**
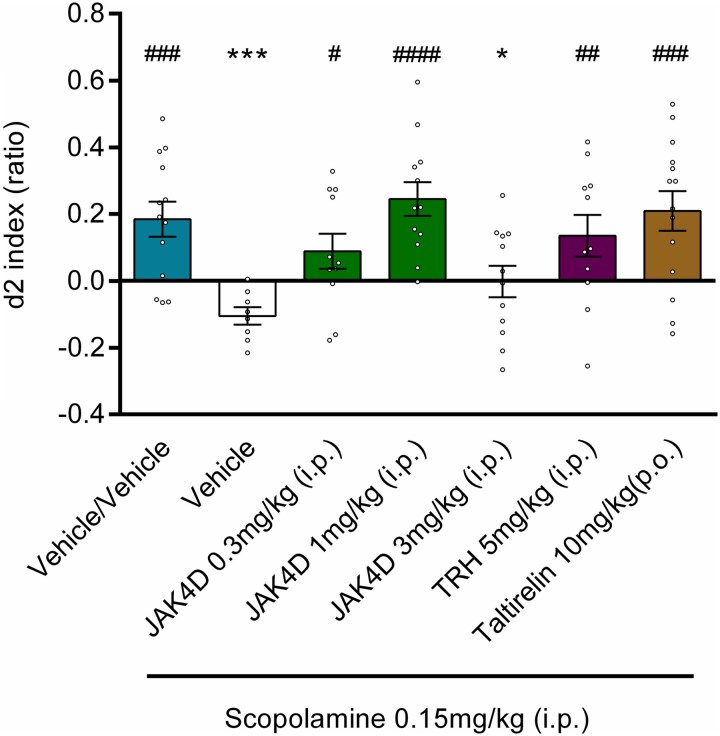
**The effect of TRH, JAK4D and taltirelin on scopolamine-induced reduction in d2 index.** Vehicle/scopolamine showed a significant decrease in d2 index in comparison to Vehicle/Vehicle. JAK4D 3 mg/kg did not reverse scopolamine induced deficit; however, JAK4D 0.3 mg/kg, JAK4D 1 mg/kg, TRH 5 mg/kg and taltirelin 10 mg/kg significantly attenuated the scopolamine-induced memory deficit when compared with Vehicle/scopolamine (*P < 0.05, ***P < 0.001 c.f. Veh/Veh & #*P* < 0.05, ##*P* < 0.01, ###*P* < 0.001, ####*P* < 0.0001 c.f. Veh/scopolamine). Values are mean ± SEM for *n* = 8–14 (see Supplementary Table 1 for each group number). *P*-values derived from ANOVA followed by least squared difference post-hoc testing. Circles represent individual d2 indices for each animal.

**Figure 2:**
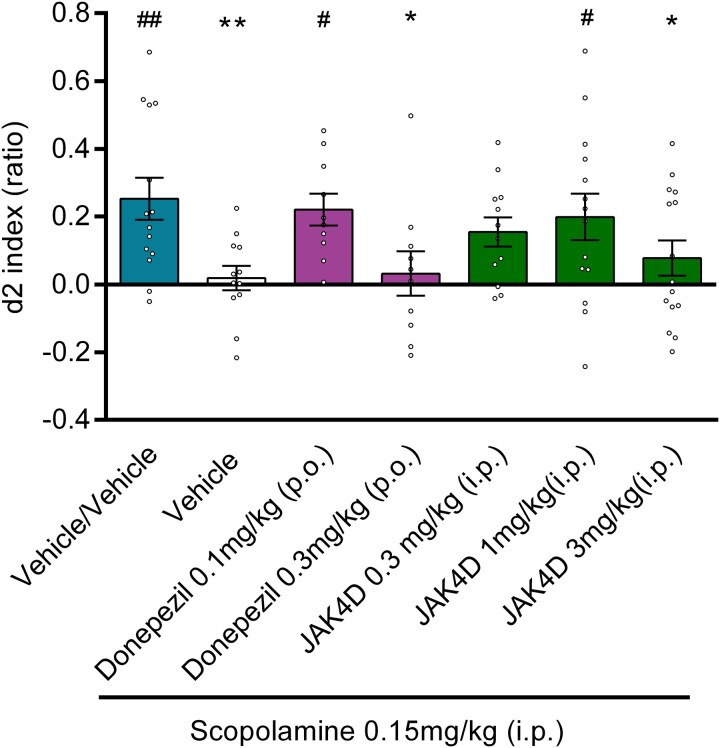
**The effect of donepezil and JAK4D on scopolamine-induced reduction in d2 index.** Vehicle/scopolamine showed a significant decrease in d2 index in comparison to Vehicle/Vehicle. Donepezil 0.3 mg/kg and JAK4D 3 mg/kg did not significantly reverse scopolamine-induced deficit; whereas donepezil 0.1 mg/kg and JAK4D 1 mg/kg showed significant increases in d2 index scores compared with Vehicle/scopolamine. (*P < 0.05, **P < 0.01 c.f. Vehicle/Vehicle, # *P* < 0.05, ##*P* < 0.01 c.f. Vehicle/scopolamine). Values are mean ± SEM for *n* = 10–15 (see Supplementary Table 3 for each group number). *P*-values derived from ANOVA followed by least squared difference post-hoc testing. Circles represent individual d2 indices for each animal.

**Figure 3:**
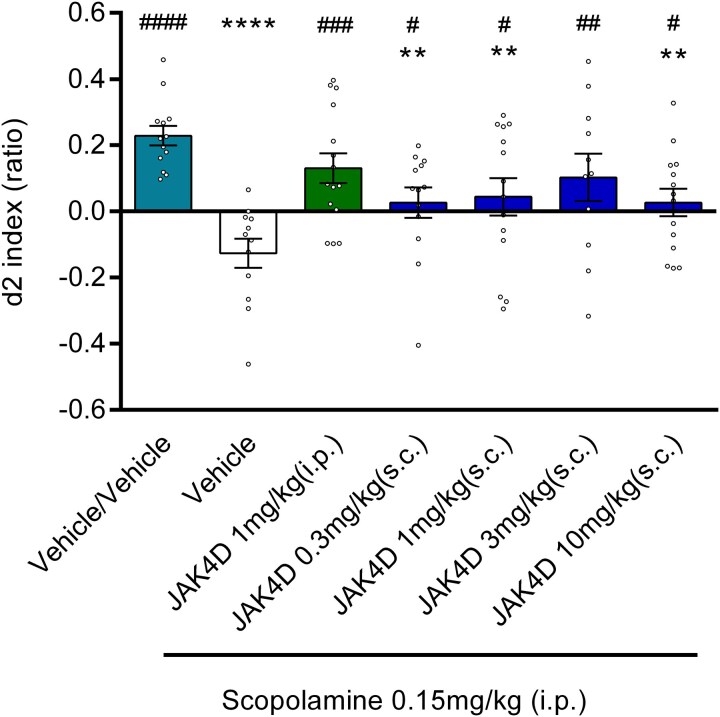
**The effect of subcutaneously and intraperitoneally administered JAK4D on scopolamine-induced reduction in d2 index.** Vehicle/scopolamine showed significant decrease in d2 index compared with Vehicle/Vehicle. All groups showed a significant increase in d2 index compared with Vehicle/scopolamine. (**P < 0.01 & ****P < 0.0001 c.f. Vehicle/Vehicle, #*P* < 0.05, ##*P* < 0.01, ###*P* < 0.001 & ####*P* < 0.001 c.f. Vehicle/scopolamine). Values are mean ± SEM for *n* = 11–15 (see Supplementary Table 5 for each group number). *P*-values derived from ANOVA followed by least squared difference post-hoc testing. Circles represent individual d2 indices for each animal.

Each animal received two injections. In brief, animals were administered scopolamine in saline or vehicle (saline only) and a test treatment or its corresponding vehicle. Scopolamine was purchased from Sigma-Aldrich (Missouri, USA). This was diluted to 0.15 ml/kg and injected intraperitoneally at 1 ml/kg in saline 30 min prior to testing (i.e. prior to the T1 trial described in the subsequent section on the NOR test) to attain an effective amnestic dose of scopolamine (0.15 mg/kg) and minimize adverse effects.^66-68^ JAK4D (Glp-Asn-Pro-DTyr-DTrpNH_2_, Part Number SP100950 Batch C, Lot number QP023087C, with 97.9% peptide purity as determined by reverse-phase HPLC) was manufactured under license by PolyPeptide Laboratories and was administered intraperitoneally (i.p.) or subcutaneously (s.c.). JAK4D was made up in dimethyl sulfoxide (DMSO)/saline (40:60) or PEG400:water:N-methyl-pyrrolidone (60:35:5) containing 5% dextrose for i.p. and s.c. administration, respectively. Each dose of JAK4D was injected at 1 ml/kg 25 min prior to testing (ptt), i.e. 5 min after scopolamine administration. TRH was made up in DMSO/saline (40:60) and administered by i.p. injection 25 min ptt (i.e. 5 min after scopolamine administration). The orally active TRH analogue, taltirelin,^59^ was purchased from Tocris Biosciences (Bristol, UK), made up in distilled water and administered orally (p.o.) 45 min ptt (i.e. 15 min prior to scopolamine administration). Donepezil was purchased from Sigma-Aldrich (Missouri, USA), made up in purified water to the required doses and administered at 1 ml/kg p.o. 60 min ppt (i.e. 30 min prior to scopolamine administration). The final administered dose for each treatment is given in Figs 1–3. Hereafter, animals who received two injections of vehicle are referred to as ‘veh/veh’, and animals who received one injection of scopolamine (0.15 mg/kg) and one injection of vehicle are referred to as ‘scop/veh’. The aim of the investigation was to assess the effect of JAK4D, TRH, taltirelin and donepezil on scopolamine-induced cognitive impairment in the NOR test rather than cognitive enhancement in healthy animals. Accordingly, the studies were run as typical designs to test the effects of procognitive drugs on scopolamine induced memory impairment, where the vehicle/vehicle and the vehicle/inducer (scopolamine in this case) groups were used for comparisons. Vehicle/test or vehicle/positive control groups were therefore not included as planned statistical comparisons were made versus the veh/veh and scop/veh groups, as described in the subsequent data and statistical analysis section.

### Study design

The study protocol, including the research question, experimental design and analysis plan, was prepared prior to commencement of the studies. Protocols were designed to minimize animal suffering and to reduce the number of animals used. As part of the study, animals were monitored for visual signs of any adverse treatment effects such as piloerection, subdued/sedation, reduced activity, hunched, abnormal breathing, mild tremors or reduced social interaction etc., that would compromise animal welfare or otherwise compromise the study parameters scored (e.g. sedative, hyperactive or anxiogenic effects that may compound NOR performance indices). The number of animals per experimental condition was based on previous testing of prospective procognitive compounds in this model by Transpharmation Limited (London, UK) (see data and statistical analysis section). In each study animals were randomly allocated to seven test groups. Study 1: Veh/Veh and Scop/Veh (*n* = 15 per group); Scop/JAK4D 0.3 mg/kg i.p.; Scop/JAK4D 1 mg/kg i.p.; Scop/JAK4D 3 mg/kg i.p.; Scop/TRH 5 mg/kg i.p. and Scop/Ceredist 10 mg/kg p.o. (*n* = 18 per group); Study 2: Veh/Veh; Scop/Veh; Scop/JAK4D 0.3 mg/kg i.p.; Scop/JAK4D 1 mg/kg i.p.; Scop/JAK4D 3 mg/kg i.p.; Scop/Donepezil 0.1 mg/kg p.o.; and Scop/Donepezil 0.3 mg/kg p.o. (*n* = 15 per group); and Study 3: Veh/Veh; Scop/Veh; Scop/JAK4D 0.3 mg/kg s.c.; Scop/JAK4D 1 mg/kg s.c.; Scop/JAK4D 3 mg/kg s.c.; Scop/JAK4D 10 mg/kg s.c. and Scop/JAK4D 1 mg/kg i.p. (*n* = 15 per group). On the day of dosing animal weights for study 1, 2 and 3 were 222–299 g, 216–264 g and 215–258 g, respectively. Treatments were balanced such that 7–8 animals had a tower as the familiar object and 7–8 had a pyramid and of each sub-group 3–4 had the novel object on the left and 3–4 had the novel object on the right. In terms of dosing, the groups were randomized throughout the day and across the four test boxes used. Following testing, animals were humanely culled by a schedule 1 method (in accordance with U.K. Scientific Procedures Act 1986).

### Habituation

The boxes used for the NOR tests were both light and sound attenuated and contained a test arena and side annex. Lighting in the boxes was set to approximately 70 lux and evening distributed throughout the box. Rats were habituated twice a day to the grey, matt, Perspex NOR box on two consecutive days prior to testing. Each habituation session consisted of a 3-min exposure to the empty test box (46 × 30 × 45 cm) followed by 1 min in the side annex (13 × 30 × 45 cm) and then a further 3 min in the test area. Prior to the second habituation session, animals were sham dosed with the appropriate vehicle (purified water, saline, DMSO/Saline (40:60) or PEG400:water: N-methyl-pyrrolidone (60:35:5) containing 5% dextrose) via the appropriate dose route according to the test group.

### Novel object recognition (NOR) test

The NOR test comprised of two sessions, T1 and T2, each lasting 3 min. JAK4D, donepezil, scopolamine, taltirelin and vehicle were administered prior to the T1 trial at the appropriate pre-testing time (see previous section on Chemicals, drug preparation and administration for details). For T1, following 3-min habituation to the empty test box, the rat was placed into the side annex and two identical objects were placed into the test area, equally spaced to each other and the two side walls. The rat was then returned to the test area and allowed to freely explore the objects for 3 min after which it was returned to its home cage. Following a 1-hour inter-trial interval, the test was repeated (T2) with one of the familiar objects substituted for a novel one of the same colour, material and size but different shape. The objects used were black pyramid and tower shapes. The protocol was the same as that for T1, i.e. 3-min habituation, 1 min in the annex while the objects were positioned followed by 3 min of exploring. Between trials the objects were cleaned with 70% ethanol to eliminate odour cues. Object exploration was recorded in both trials (T1 and T2). Recall in T2 is dependent on the rat’s ability to remember the objects presented during T1 and characteristically the animal demonstrates increased exploration of the novel object following recognition of the familiar object.

### Scoring

Trials were recorded by GeoVision surveillance camera software and files were saved on portable solid state hard drives for remote scoring. Exploration was scored by an observer blind to study treatments and the novel object as time spent sniffing or licking the object, when the nose was in contact with the object and moving (i.e. when the animal was sniffing). Sitting on the object or next to it with the nose directed away was not classed as exploration. The performance measures were as follows:

T1 (learning trial time) = total time exploring both objects in T1. The T1 score provides an indication whether the compound is affecting overall exploration (e.g. sedative, hyperactive, anxiogenic), which may then confound the NOR performance indices.

T2 (test trial time) = total time exploring both objects in T2.

d1 (difference score) = time spent exploring novel object—time spent exploring familiar object

d2 (relative difference score) = $\frac{d1}{T2}$

The d2, or discrimination, index is the most commonly used measure of object recognition, which is not influenced by differences in exploration time and represents the time exploring the novel object minus the time exploring the familiar object, divided by the total exploration time.^65,67^ The d2 index provides a standardized method of object recognition memory in the NOR test and is used herein as the primary outcome measure.

### Data and statistical analysis

Predefined exploration measures (presented in the scoring section above) and exclusion criteria were employed, as described by others.^62,66,68^ Animals showing a lack of exploration activity or interest in the task were excluded utilizing the predefined exclusion criteria in order to ensure animals included in data analyses were adequately engaging with the task. Thus, animals were excluded from data analyses if the T1 score failed to reach the minimum of 15 s of exploration, the T2 score failed to reach the minimum 10 s of exploration. Also, animals were excluded if they experienced adverse effects, e.g. scopolamine-related breathing issues (dyspnoea) or if they were statistical outliers, i.e. over 2-fold greater than SD values. Details of animals excluded from statistical analyses in each of the studies are provided in Supplementary Tables 1, 3 and 5. Power analyses were run by Transpharmation on internal NOR studies to determine the sample size required for 80% power and 5% significance level for the d2 index, which has consistently given a result of *n* = 13–15. The group size of 15 was chosen to account for possible non-explorers (as those that explore less than 10 s are excluded from the analysis).

The T1, T2, d1 and d2 scores were analysed using a two-way analysis of variance (ANOVA, object × treatment) followed by planned comparisons post-hoc least significant difference using single measure parametric analysis, based on least square (predicted) means in Prism (GraphPad Inc., MA, USA) from which t-statistics, *P* values and degrees of freedom (DF) for all comparisons were determined. Planned comparisons were made versus veh/veh and scop/veh groups. In the analysis, ‘object’ is included as a factor to evaluate potential object bias. Sizes of treatment effects upon d2 scores, relative to control (either scop/veh or veh/veh) treatment, were determined by Cohen’s *d*,^69^ calculated as:


$$d=\frac{{\bar{x}}_{scop/treatment}-{\bar{x}}_{control}}{\sqrt{MSE}}$$

Where ${\bar{x}}_{scop/treatment}$ is the mean d2 value of the group treated with scopolamine and the treatment of interest, ${\bar{x}}_{control}$ is the mean d2 value of the scop/veh or veh/veh-treated group. The MSE is the mean squared error calculated across all groups within the two-way ANOVA, such that effect sizes correspond to the *P*-values calculated by Fishers least significant difference posthoc testing. Data are expressed as mean ± standard error of the mean (SEM) and are considered significant when *P* < 0.05.

## Results

### Scopolamine-induced deficits in NOR task performance

Across all tests, scopolamine HBr at 0.15 mg/kg i.p. produced a pharmacological deficit in the rat NOR memory test (Figs 1–3). This was evidenced by a significant reduction in d2 index with scopolamine treatment compared to the vehicle-treated controls (study 1: *t* = 3.6, DF = 67, *P* = 0.0006, *d* = −1.68, study 2: *t* = 2.9, DF = 73, *P* = 0.0047, *d* = −1.14, study 3: *t* = 5.1, DF = 78, *P* < 0.0001, *d* = −2.08) reflecting decreased exploration of the novel object relative to the familiar object in the NOR test. The scopolamine-induced deficit did not affect T1 or T2 exploration scores in study 1 and 2 (Supplementary Tables 1 and 2, and 3 and 4, respectively). A small but significant decrease in T1 (but not T2) score was observed in study 3 for scop/veh compared to veh/veh (Supplementary Tables 5 and 6). A decrease in T1 only has potential to increase d2 index. As such, difference in T1 score could not be the basis of the observed decrease in d2 index in scop/veh compared to veh/veh observed in study 3. Thus, all test studies were considered valid.

### Study 1: effect of JAK4D, TRH and taltirelin on scopolamine-induced memory impairment

Results of study 1 are illustrated in Fig. 1. The scopolamine-induced performance deficit in the rat NOR test was significantly reversed (i.e. no significant difference versus veh/veh, significant increase versus scop/veh) by both TRH (5 mg/kg i.p., administered 5 min after scopolamine: versus veh/veh *t* = 0.67, *P* = 0.5026, *d* = −0.28, versus scop/veh *t* = 2.9, *P* = 0.0055, *d* = 1.40), and taltirelin (10 mg/kg p.o., administered 15 min prior to scopolamine: versus veh/veh *t* = 0.35, *P* = 0.7238, *d* = 0.11, versus scop/veh *t* = 3.9, *P* = 0.0002, *d* = 1.79). JAK4D (0.3–1 mg/kg i.p., administered 5 min following scopolamine) significantly attenuated the memory deficit in a dose-dependent manner, with animals treated with these doses showing no significant difference in d2 index to vehicle-treated animals (0.3 mg/kg: versus veh/veh *t* = 1.3, *P* = 0.1979, *d* = −0.56, versus scop/veh *t* = 2.3, *P* = 0.0234, *d* = 1.12, 1 mg/kg: versus veh/veh *t* = 0.84, *P* = 0.4055, *d* = 0.33, versus scop/veh *t* = 4.3, *P* < 0.0001, *d* = 2.01). However, the highest dose of JAK4D (3 mg/kg) did not attenuate the memory deficit (Fig. 1, versus veh/veh *t* = 2.6, *P* = 0.0118, −1.06, versus scop/veh *t* = 1.3, *P* = 0.2133, *d* = 0.61), indicative of an inverted U-shaped dose response. For all statistics, DF = 67. T1 and T2 exploration scores were not significantly affected by JAK4D or taltirelin when compared with vehicle-treated controls; however, a small but significant decrease in T1 exploration was observed for TRH (Supplementary Table 1). ANOVA results for each performance statistic for study 1 are listed in Supplementary Table 2.

### Study 2: effect of JAK4D and donepezil on scopolamine-induced recognition memory deficit

Results of study 2 are illustrated in Fig. 2. The scopolamine-induced deficit was again found to be significantly and fully reversed by JAK4D (1 mg/kg i.p., administered 5 min following scopolamine, versus Veh/veh *t* = 0.69, *P* = 0.4909, *d* = −0.25, versus Scop/veh *t* = 2.3, *P* = 0.0274, *d* = 0.89), with treated animals showing no significant difference to veh/veh-treated animals. The degree of reversal was similar to that of donepezil at 0.1 mg/kg p.o. (versus Veh/veh *t* = 0.43, *P* = 0.6720, *d* = −0.15, versus Scop/veh *t* = 2.3, *P* = 0.0263, *d* = 0.99). For all statistics, DF = 73. No clinical limiting treatment effects or adverse effects (described in the study design) that could affect NOR performance indices were observed in animals treated with either donepezil or JAK4D. T1 and T2 exploration scores were not significantly affected by JAK4D (at any of the test doses) or donepezil (at any of the test doses) when compared with vehicle-treated controls (Supplementary Table 3). ANOVA results for each performance statistic for study 2 are listed in Supplementary Table 4.

### Study 3: comparison of JAK4D administered via intraperitoneal and subcutaneous routes in reversal of scopolamine-induced deficits

Results of study 3 are illustrated in Fig. 3. The effect of subcutaneously administered JAK4D in scopolamine deficit NOR test was compared to the maximally effective dose observed for i.p. injection of JAK4D. Concurring with the results obtained in Studies 1 and 2, the scopolamine-induced deficit was significantly reduced by i.p. administration of JAK4D (1 mg/kg, administered 5 min following scopolamine), with treated animals displaying d2 scores comparable to the veh/veh group (versus veh/veh *t* = 1.5, *P* = 0.1365, *d* = −0.58, versus scop/veh *t* = 3.8, *P* = 0.0002, *d* = 1.50). Subcutaneous administration of JAK4D (0.3–10 mg/kg, administered 5 min following scopolamine) produced an inverse U-shaped dose–response behavioural profile. JAK4D (0.3–10.0 mg/kg s.c.) showed a significant (*P* < 0.05) dose-related reversal of scopolamine-induced memory deficit with a maximal effect at 3.0 mg/kg JAK4D (0.3 mg/kg: versus veh/veh *t* = 3.2, *P* = 0.0023, *d* = −1.21, versus scop/veh *t* = 2.1, *P* = 0.0432, *d* = 0.87, 1 mg/kg: versus veh/veh *t* = 2.8, *P* = 0.0067, *d* = −1.10, versus scop/veh *t* = 2.5, *P* = 0.0141, *d* = 0.98, 3 mg/kg: versus veh/veh *t* = 1.8, *P* = 0.0788, *d* = −0.75, versus scop/veh *t* = 3.2, *P* = 0.0021, *d* = 1.32, 10 mg/kg: versus veh/veh *t* = 3.0, *P* = 0.0032, *d* = −1.15, versus scop/veh *t* = 2.3, *P* = 0.0267, *d* = 0.92). At 3 mg/kg s.c., JAK4D exhibited full reversal of the scopolamine-induced deficit, with treated animals displaying no difference from the veh/veh-treated controls. For all statistics, DF = 78.

T1 and T2 exploration scores were not significantly affected by JAK4D (1 mg/kg i.p.) or JAK4D (3 mg/kg s.c.) when compared with veh/veh. A reduction in T1 exploration was observed in the s.c. JAK4D groups (0.3, 1 and 10 mg/kg) compared to veh/veh (Supplementary Table 5). However, this decrease was not significant compared with the scop/veh group (the reference, which also showed significantly lower T1 compared to veh/veh). As such, this is not considered to impact interpretation of the effect of JAK4D on d2 index. ANOVA results for each performance statistic for study 3 are listed in Supplementary Table 6.

## Discussion

The work of others demonstrates that the scopolamine challenge test disrupts cholinergic signalling and simulates clinically relevant changes in neuronal networking and cognitive functioning.^14-17,23^ As such, it provides a valuable pharmacological model for the assessment of new prospective pro-cognitive drugs. Consistently, across all investigations in this study, treatment with JAK4D (1 mg/kg i.p.) significantly reversed the effects of scopolamine—with animals administered JAK4D after scopolamine showing similar NOR task performance to vehicle-treated animals.

The effects of scopolamine on task performance were comparably reversed by JAK4D (1 mg/kg i.p.), TRH (5 mg/kg i.p.) and taltirelin (10 mg/kg p.o.). The effect of TRH on scopolamine-induced cognitive deficit in the rat has been shown to translate to humans where TRH (5 mg/kg i.v.) reverses memory impairment in young healthy volunteers pretreated with scopolamine (0.5–0.75 mg i.v.).^50^ In young healthy volunteers, scopolamine induces a pattern of memory and cognitive deficits that is markedly similar to the changes occurring in mild Alzheimer’s disease patients and the scopolamine challenge test in these subjects is considered to provide a model of the core deficits of Alzheimer’s disease.^70^ In elderly volunteers, the effects of scopolamine on cognition are more pronounced than in young healthy volunteers, likely reflecting the compounding of muscarinic antagonism with additional central cholinergic systems deficits associated with ageing.^71-73^ Consequently, the cognitive deficits of scopolamine-treated elderly volunteers are not considered to constitute a valid model of Alzheimer’s disease.^74^ In this regard, in an additional study in older volunteers (aged 65 and over), TRH did not attenuate scopolamine-induced cognitive impairment.^75^ Importantly, supporting clinical translatability, TRH (0.3 mg/kg i.v. infusion) increases arousal and improves affect, as well as semantic memory in people with Alzheimer’s disease,^51^ despite its pharmacological limitations.

The observed effect of taltirelin on scopolamine-induced deficit in the rat NOR test is consistent with previous data reported by,^76^ which showed that orally administered taltirelin treatment had a significant effect in scopolamine-treated rats on the delayed alternation task (T-maze)—a spatial working memory task. Our finding that JAK4D elicits comparable procognitive effects to TRH and taltirelin, alongside our previous demonstration that JAK4D does not interact with the pituitary TRH receptors,^55^ provides further evidence that JAK4D can be employed to harness the central therapeutic actions of TRH while avoiding undesirable off-target endocrine actions. Notably, these results collectively build out and underscore the relevance of central TRH signalling system activation for the treatment of cognitive deficits in patients with neurodegenerative diseases.

Advantageously, taltirelin can be administered orally.^59^ Oral delivery is the most preferred route of drug administration due in part to convenience and improved patient compliance. In general, however, unfavourable physicochemical properties prevent successful oral peptide delivery^77^ and the parenteral route of delivery remains the predominant route of administration for therapeutic peptides.^78^ To explore potential utility of s.c. administration, we compared the effects on scopolamine-induced performance deficit of ascending doses of JAK4D given by s.c. injection (0.3, 1, 3 and 10 mg/kg) to those of the neuropharmacologically active dose of 1 mg/kg i.p. Concurring with studies 1 and 2, the positive control JAK4D (1 mg/kg i.p.) significantly and fully reversed the scopolamine-induced deficit. Subcutaneously administered JAK4D (0.3–10 mg/kg) also showed a significant dose-related reversal of scopolamine-induced memory deficit, as measured by the d2 index, with a maximal effect at 3.0 mg/kg resulting in comparable performance to non-challenged rodents. The pro-cognitive signal observed for JAK4D in this scopolamine challenge model indicates that this first-in-class TRH-based compound is CNS penetrant and can achieve suitable CNS bioavailability in rat when administered parenterally. This concurs with previous studies in mouse showing that systemically administered JAK4D is able to penetrate the blood–brain barrier and reach target TRH receptors in the brain at appropriate levels for central TRH receptor activation.^55^

The inverted U-shaped dose–response curves observed for both i.p. and s.c. administered JAK4D are a common feature of cognitive enhancers in memory and learning studies.^79-81^ This hormetic effect, whereby too high or too low a dose leads to a suboptimal or no response has been reported for cognitive enhancers with different mechanisms of action, including donepezil (Aricept), tacrine, rivastigmine, galantamine, memantine and the 5-HT6 agonists, E-6801 and EMD-386088.^79,81-84^ Such an effect may reflect that cognitive and behavioural performance in models of memory and learning operate most effectively within an optimal activity range, such that too much or too little neuromodulation can impair performance.^83,85^ Biphasic dose–response relationships characterized by low-dose stimulation and high-dose inhibition commonly occur throughout biological sciences and clinical medicine and have been discussed extensively by Calabrese and colleagues, for example, see Calabrese and Mattson, 2017.^86^ Hormesis may be underpinned by adaptive responses to compensate for homeostatic imbalance. In this regard, the inverted U shape dose–response curves observed for cholinesterase inhibitors, it is suggested that high levels of acetylcholine may trigger activation of presynaptic muscarinic autoreceptors to limit acetylcholine released by cholinergic neurons, thereby contributing to a consequent reduction in cognitive functioning.^82^ Stimulation of cholinergic pathways via activation of central TRH signalling system may similarly be reflected in the inverted U-shaped dose–response curves observed for JAK4D. The inverted dose–response relationship has been observed in the clinical use of cholinesterase inhibitors^82^; however, it has been suggested that this effect is less evident in clinical trials.^87^ In pharmaceutical development, understanding dose response is important for dose selections in early clinical trials. The Bayesian Emax model, which assumes the drug effect is proportional to the dose, i.e. the bigger the dose, the bigger the effect, is commonly used to facilitate dose selection. In the case of non-monotonic U-shaped dose–response relationships, however, such as may be observed with cholinergic stimulation, the normal dynamic linear model method model may provide a more useful model to assess dose response, as has been demonstrated for the rheumatoid arthritis drug candidate GSK654321.^88^

Acetylcholine esterase inhibitors, such as donepezil, are designed to address cholinergic system dysfunction, which is a hallmark of cognitive impairment across a range of neurodegenerative diseases,^2,18,85,89,90^ by bolstering declining levels of acetylcholine through inhibition of its breakdown. This therapeutic approach has been used to attenuate cognitive decline and is symptomatic treatment for cognitive impairment in Alzheimer’s disease^2,91^ and Parkinson’s disease.^92^ However, clinical benefit of this class of drugs is limited by peripherally mediated side effects and by the loss of cholinergic neurons as disease progresses.

Restoration of performance in the NOR test by JAK4D (1 mg/kg i.p.) was similar to that observed for donepezil (0.1 mg/kg p.o.). Therefore, in this model, JAK4D provides similar therapeutic effects against memory impairment to this gold-standard treatment of Alzheimer’s disease. Given the clinical translatability of the scopolamine challenge rodent model, and the established therapeutic effect of donepezil against memory impairment in Alzheimer’s disease, our findings of comparable effects by JAK4D support its development as a potential therapeutic for Alzheimer’s disease and other neurocognitive disorders.

Current perspectives are rekindling a renewed focus on the role of cholinergic system dysfunction in the pathophysiology of cognitive impairment and on the development of next generation pro-cholinergic interventions.^2,93-95^ Ideally, neuropharmacological interventions that can potentiate cholinergic signalling and also modulate underlying pathophysiological processes driving progressive neuronal loss are needed to improve cognitive function and slow disease progression in Alzheimer’s disease and related diseases. Advantageously, JAK4D has therapeutic potential to slow disease progression, in addition to reducing cognitive impairment, by protecting against neuronal loss and counteracting core pathophysiological processes driving cell death—i.e. excessive reactive oxygen species and excitotoxicity.^55^

Neuropeptides, such as TRH, are pleiotropic state-dependent neuromodulators and important homeostatic regulators of neuronal circuit function.^33,96-100^ As a drug class they have undoubted advantages, including high potency and diverse clinically relevant neuropharmacological effects; however, the rapid degradation of natural endogenous neuropeptides presents a challenge to harnessing their clinical potential.^101-103^ As exemplified by degradation-stabilized GLP-1 receptor agonists, the invention of degradation resistant peptide analogues is pivotal to unlocking their therapeutic potential and widening appreciation of the broad clinical potential of neuropeptide signalling systems.^54,101,104,105^ Potent inhibition of TRH-DE by JAK4D may be conjectured to increase local endogenous concentrations of TRH in the pituitary with consequent TRH-induced stimulation of TSH release. However, anterior pituitary basal levels of TRH-DE are very low in comparison to CNS tissues and evidence indicates that anterior pituitary TRH-DE activity has minimal influence on TSH secretion.^52^  ^,106-108^ Accordingly, JAK4D (1 mg/kg administered to male Wistar rats via i.p. injection) is not observed to significantly increase the release of TSH *in vivo.*^61^ Thus, with regard to TRH, the innovative design of JAK4D not only overcomes the problems of rapid degradation in brain and serum but also provides a means to deliver the multifactorial neurotherapeutic actions of central TRH receptor activation without inducing endocrine side effects.

The scopolamine induced amnesia model is an acute pharmacologically induced model of cognitive impairment; hence, present data relate to an acute model of cholinergic dysfunction rather than a chronic neurodegenerative disease model. Therefore, a limitation of the present study is that the animals tested in this model are not inherently impaired in cognition and do not represent a model of progressive cognitive decline. In common with other neuropharmacological models, the scopolamine challenge test does not fully reflect the complexity of human neurodegenerative diseases, such as AD, but rather captures specific domains of pathophysiology and provides a means to screen for drug candidates that can mitigate the effects of specific disease associated mechanisms. While scopolamine is recognized to be an amnesic drug that disrupts central cholinergic neurotransmission, an acknowledged limitation of this neuropharmacological agent is that it lacks selectivity for central muscarinic receptors and can influence noncognitive aspects of behaviour and affect peripheral, physiological processes, particularly at high doses.^109,110^ Thus, the model developed at Transpharmation and employed in the current studies utilizes the lowest possible dose of scopolamine consistently and reliably shown to induce memory deficit in the NOR test with Lister Hooded rats while minimizing adverse peripheral effects. The limitations of the scopolamine model, as well as the advantages and translatability of this model, have been extensively reviewed in a recent paper.^111^ In concluding, Jagielska *et al*.,^111^ note that, although careful consideration needs to be given to the experimental design to mitigate adverse effects, the scopolamine model represents a valuable tool for assessing prospective precognitive compounds. Since a single model cannot wholly capture the human condition, efficacy in more than one clinically-relevant model is recognized to increase confidence in the translation potential of a drug candidate.^55^ Notably, data from the scopolamine challenge model provide further evidence that JAK4D can counteract pathophysiological mechanisms underlying neurodegenerative disease-related cognitive deficits.

## Conclusions

The reversal of scopolamine-induced cognitive deficits by JAK4D in the current behavioural pharmacology study using the NOR task support the pro-cognitive potential of JAK4D under conditions of cholinergic dysfunction. The results of the current study taken together with the positive therapeutic benefits observed in earlier studies, which among other things show JAK4D is neuroprotective and significantly reduces hippocampal-dependent memory deficits,^55^ demonstrate the potential of JAK4D to mitigate cognitive impairment in patients with neurodegenerative diseases.

## Supplementary Material

fcag006_Supplementary_Data

## Data Availability

Data generated in this study are available from the corresponding author upon reasonable request.
