# Supplementary material for: JAK4D, a first-in-class thyrotropin-releasing hormone analogue, reverses scopolamine-induced memory deficits
Source: Brain Commun. 2026 Jan 16;8(1):fcag006. doi: 10.1093/braincomms/fcag006 (PMC12848665; doi:10.1093/braincomms/fcag006)
Supplement: fcag006_Supplementary_Data [file fcag006_supplementary_data.pdf]

Treatment (mg/kg)	Time exploring novel object (s)	Time exploring familiar object (s)	d1 Index (s)	d2 Index (s)	T1 score (s)	T2 score (s)	n
Veh/Veh	15.42 ± 1.69	11.04 ± 1.61	4.38 ± 1.59 ##	0.19 ± 0.05 ###	38.22 ± 4.49	26.47 ± 2.89	13
Scop/Veh	11.8 ± 1.09	14.65 ± 1.47	-2.85 ± 0.87 **	-0.11 ± 0.03 ***	41.55 ± 3.64	26.45 ± 2.44	8
Scop/JAK4D 0.3 (i.p.)	16.28 ± 2.91	13.42 ± 1.94	2.86 ± 1.80 #	0.09 ± 0.05 #	38.23 ± 4.58	29.70 ± 4.60	11
Scop/JAK4D 1 (i.p.)	15.90 ± 1.64	10.18 ± 1.65	5.72 ± 1.17 ###	0.25 ± 0.05 ####	33.77 ± 3.94	26.08 ± 3.07	12
Scop/JAK4D 3 (i.p.)	12.30 ± 1.46	12.85 ± 1.72	-0.56 ± 1.04 *	0.00 ± 0.05 *	40.14 ± 4.74	25.15 ±3.02	12
Scop/TRH 5 (i.p.)	14.28 ± 1.88	11.03 ± 1.47	3.25 ± 1.34 ##	0.14 ± 0.06 ##	27.89 ± 3.25 #	25.31 ± 3.10	11
Scop/Taltirelin 10 (p.o.)	11.89 ± 1.33	8.60 ± 1.02	4.52 ± 1.50 ###	0.21 ± 0.06 ###	35.80 ± 3.22	20.49 ± 1.61	14

Supplementary Table 1. Study 1: The effect of TRH, JAK4D and taltirelin on scopolamine-induced deficit in the rat NOR test. The table shows the time spent exploring the novel and familiar objects during T2 and total time exploring two objects during T1 (T1 score) and T2 (T2 score), as well as values for d1 and d2 indexes. Animals (numbers in brackets) were excluded from statistical analyses from the following groups as: (a) the T2 score failed to reach the minimum of 10 s: Veh/Veh (1), Scop/Veh (5), Scop/JAK4D 0.3 mg/kg (5), Scop/JAK4D 1 mg/kg (4), Scop/JAK4D 3 mg/kg (2), Scop/TRH (6), and Scop/Taltirelin (4); (b) the T1 score failed to reach the minimum of 15 s: Scop/Veh (2), Scop/JAK4D 0.3 mg/kg (1), Scop/JAK4D 1 mg/kg (1), Scop/JAK4D 3 mg/kg (2); (c) animals were culled due to adverse effects related to scopolamine: Scop/JAK4D 0.3 mg/kg (1), Scop/JAK4D 3 mg/kg (1); and (d) animals were statistical outliers (over 2-fold > SD value): Veh/Veh (1), Scop/JAK4D 1 mg/kg (1), Scop/JAK4D 3 mg/kg (1), Scop/TRH (1). Data are expressed as means ± s.e.m. n=8-14. *P<0.05, **P<0.01, ***P<0.001 c.f. Veh/Veh. #P<0.05, ##P<0.01, ###P<0.001, ####P<0.0001 c.f. Veh/Scop. Values are mean ± SEM n=8-14. P-values are derived from ANOVA followed by post-hoc least squared difference tests.

	SS	DF	MS	F (DFn, DFd)	P value
**T1 score**					
Interaction	848.1	6	141.4	F (6, 66) = 0.7325	0.6252
Row Factor	1461	6	243.5	F (6, 66) = 1.262	0.2870
Column Factor	168.2	1	168.2	F (1, 66) = 0.8717	0.3539
Residual	12736	66	193.0		
**T2 score**					
Interaction	379	6	63	F (6, 67) = 0.58	0.7464
Row Factor	767	6	128	F (6, 67) = 1.2	0.3330
Column Factor	257	1	257	F (1, 67) = 2.3	0.1300
Residual	7326	67	109		
**d1 score**					
Interaction	230	6	38	F (6, 67) = 1.7	0.1247
Row Factor	559	6	93	F (6, 67) = 4.2	0.0011
Column Factor	22	1	22	F (1, 67) = 1.0	0.3152
Residual	1472	67	22		
**d2 score**					
Interaction	0.29	6	0.048	F (6, 67) = 1.5	0.1930
Row Factor	0.92	6	0.15	F (6, 67) = 4.8	0.0004
Column Factor	0.0041	1	0.0041	F (1, 67) = 0.13	0.7230
Residual	2.2	67	0.032		

Supplementary Table 2. Study 1: two-way ANOVA table for T1, T2, d1 and d2 scores.SS – Sum of Squares. DF – Degrees of freedom. MS – Mean squares. DFn – Numerator degrees of freedom. DFd – Denominator degrees of freedom. Column - Object category, Row factor - Treatment category.

Treatment (mg/kg)	Time exploring novel object (s)	Time exploring familiar object (s)	d1 Index (s)	d2 Index (s)	T1 score (s)	T2 score (s)	n
Veh/Veh	13.64 ± 1.32	8.22 ± 1.06	5.42 ± 1.52 #	0.25 ± 0.06 ##	29.89 ± 1.42	21.86 ± 1.85	14
Scop/Veh	12.40 ± 1.17	11.77 ± 0.90	0.64 ± 0.97 *	0.02 ± 0.04 **	31.85 ± 1.34	24.17 ± 1.85	12
Scop/JAK4D 0.3 i.p.	15.32 ± 1.61	11.14 ± 1.38	3.88 ± 1.09	0.15 ± 0.04	32.21 ± 1.71	25.17 ± 2.80	12
Scop/JAK4D 1 (i.p.)	13.46 ± 1.48	9.72 ± 1.62	3.74 ± 1.35	0.20 ± 0.07 #	29.38 ± 1.61	23.18 ± 2.79	14
Scop/JAK4D 3 (i.p.)	10.96 ± 0.93	9.37 ± 0.81	1.59 ± 1.13 *	0.08 ± 0.05 *	27.33 ± 1.46	20.34 ± 1.33	15
Scop/Donepezil 0.1 (p.o.)	15.85 ± 2.24	9.91 ± 1.10	6.00 ± 1.68 ##	0.22 ± 0.05 #	28.49 ± 2.64	25.76 ± 3.12	10
Scop/Donepezil 0.3 (p.o.)	11.09 ± 1.34	11.28 ± 2.04	-0.19 ± 1.16 **	0.03 ± 0.07 *	31.17 ± 2.11	22.37 ± 3.24	10

Supplementary Table 3. Study 2: The effect of donepezil and JAK4D on scopolamine-induced deficit in the rat NOR test. The table shows the time spent exploring the novel and familiar objects during T2 and total time exploring two objects during T1 (T1 score) and T2 (T2 score), as well as values for d1 and d2 indexes. Animals (numbers in brackets) were excluded from statistical analyses from the following groups as: (a) the T2 score failed to reach the minimum of 10 s: Veh/Veh (1); Scop/Veh (1), Scop/JAK4D 0.3 mg/kg (2), Scop/Donepezil 0.1 mg/kg (3), and Scop/Donepezil 0.3 mg/kg (3); (b) the T1 score failed to reach the minimum of 15 s: Scop/JAK4D 1 mg/kg (1), Scop/Donepezil 0.1 mg/kg (1); (c) animals were culled due to adverse effects related to scopolamine: Scop/JAK4D 0.3 mg/kg (1), Scop/Donepezil 0.3 mg/kg (1); and (d) animals were statistical outliers (over 2-fold > SD value): Scop/Veh/ (2), Scop/Donepezil 0.1 mg/kg mg/kg (1), Scop/Donepezil 0.3 mg/kg (1). Data are expressed as means ± s.e.m. n=10-15. *P<0.05, **P<0.01 c.f. Veh/Veh. #P<0.05, ##P<0.01c.f. Veh/Scop. P-values are derived from ANOVA followed by post-hoc least squared difference tests.

	SS	DF	MS	F (DFn, DFd)	P value
**T1 score**					
Interaction	124	6	21	F (6, 73) = 0.55	0.7712
Row Factor	248	6	41	F (6, 73) = 1.1	0.3751
Column Factor	12	1	12	F (1, 73) = 0.32	0.5753
Residual	2757	73	38		
**T2 score**					
Interaction	344	6	57	F (6, 73) = 0.78	0.5886
Row Factor	252	6	42	F (6, 73) = 0.57	0.7525
Column Factor	11	1	11	F (1, 73) = 0.15	0.7007
Residual	5376	73	74		
**d1 score**					
Interaction	57	6	9.5	F (6, 73) = 0.43	0.8540
Row Factor	356	6	59	F (6, 73) = 2.7	0.0193
Column Factor	32	1	32	F (1, 73) = 1.4	0.2332
Residual	1592	73	22		
**d2 score**					
Interaction	0.022	6	0.0037	F (6, 73) = 0.090	0.9972
Dose group	0.59	6	0.099	F (6, 73) = 2.4	0.0365
T1 object	0.056	1	0.056	F (1, 73) = 1.4	0.2481
Residual	3.0	73	0.041		

Supplementary Table 4. Study 2: two-way ANOVA table for T1, T2, d1 and d2 scores. SS – Sum of Squares. DF – Degrees of freedom. MS – Mean squares. DFn – Numerator degrees of freedom. DFd – Denominator degrees of freedom. Column - Object category, Row factor - Treatment category.

Treatment (mg/kg)	Time exploring novel object (s)	Time exploring familiar object (s)	d1 Index (s)	d2 Index (s)	T1 score (s)	T2 score (s)	n
Veh/Veh	14.86 ± 1.18	9.32 ± 0.81	5.54 ± 0.81 ####	0.23 ± 0.03 ####	36.37 ± 2.45 #	24.17 ± 1.86	13
Scop/Veh	9.31 ± 1.21	12.15 ± 1.42	-2.84 ± 1.06 ****	-0.13 ± 0.04 ****	28.63 ± 3.23 *	21.45 ± 2.42	12
Scop/JAK4D 0.3 (s.c.)	11.51 ± 1.27	11.23 ± 1.20	0.27 ± 1.30 **	0.02 ± 0.05 # **	27.12 ± 2.41 **	22.74 ± 2.11	13
Scopo/JAK4D 1 (s.c.)	10.62 ± 0.91	11.14 ± 1.79	-0.53 ± 1.34 ***	0.04 ± 0.06 # **	28.52 ± 2.76 *	21.76 ± 2.50	14
Scop/JAK4D 3 (s.c.)	13.39 ± 1.62	11.84 ± 1.83	2.29 ± 1.67 ##	0.10 ± 0.07 ##	30.81 ± 1.76	25.24 ± 3.03	11
Scop/JAK4D 10 (s.c.)	11.22 ± 1.06	10.73 ± 1.00	0.54 ± 0.99 # **	0.03 ± 0.04 # **	29.42 ± 2.64 *	21.77 ± 1.81	14
Scop/JAK4D 1(i.p.)	13.03 ± 1.25	10.18 ± 1.18	2.85 ± 1.02 ###	0.13 ± 0.05 ###	30.96 ± 1.92	23.21 ± 2.21	15

Supplementary Table 5. Study 3: The effect of subcutaneously- and intraperitoneally-administered JAK4D on scopolamine-induced deficit in the rat NOR test. The table shows the time spent exploring the novel and familiar objects during T2 and total time exploring two objects during T1 (T1 score) and T2 (T2 score), as well as values for d1 and d2 indexes. Animals (numbers in brackets) were excluded from statistical analyses from the following groups as: (a) the T2 score failed to reach the minimum of 10 s: Veh/Veh (1), Scop/Veh (2), Scop/JAK4D 0.3 mg/kg (1), Scop/JAK4D 1 mg/kg s.c. (1), Scop/JAK4D 3 mg/kg s.c. (4), Scop/JAK4D 10 mg/kg s.c. (1), (b) the T1 score failed to reach the minimum of 15 s: none; (c) animals were culled due to adverse effects related to scopolamine: Scop/JAK4D 0.3 mg/kg s.c. (1); and (d) animals were statistical outliers (over 2-fold > SD value): Veh/Veh (1), Scop/Veh/ (1. Data are expressed as means ± s.e.m. n=11-15. *P<0.05, **P<0.01, ***P<0.001, ****P<0.0001 c.f. Veh/Veh. #P<0.05, ##P<0.01, ###P<0.001, ####P<0.0001 c.f. Veh/Scop. P-values are derived from ANOVA followed by post-hoc least squared difference tests.

	SS	DF	MS	F (DFn, DFd)	P value
**T1 score**					
Interaction	313.0	6	52.17	F (6, 78) = 0.6583	0.6833
Row Factor	544.3	6	90.72	F (6, 78) = 1.145	0.3447
Column Factor	493.6	1	493.6	F (1, 78) = 6.229	0.0147
Residual	6181	78	79.25		
**T2 score**					
Interaction	857	6	143	F (6, 78) = 2.4	0.0377
Row Factor	103	6	17	F (6, 78) = 0.28	0.9431
Column Factor	280	1	280	F (1, 78) = 4.6	0.0344
Residual	4714	78	60		
**d1 score**					
Interaction	157	6	26	F (6, 78) = 1.5	0.2011
Row Factor	557	6	93	F (6, 78) = 5.2	0.0001
Column Factor	0.26	1	0.26	F (1, 78) = 0.015	0.9036
Residual	1391	78	18		
**d2 score**					
Interaction	0.31	6	0.052	F (6, 78) = 1.7	0.1244
Row Factor	0.88	6	0.15	F (6, 78) = 4.9	0.0003
Column Factor	$2.2*10^{-5}$	1	$2.2*10^{-5}$	F (1, 78) = $7.5*10^{-4}$	0.9782
Residual	2.3	78	0.030		

Supplementary Table 6. Study 3: two-way ANOVA table for T1, T2, d1 and d2 scores. SS – Sum of Squares. DF – Degrees of freedom. MS – Mean squares. DFn – Numerator degrees of freedom. DFd – Denominator degrees of freedom. Column - Object category, Row factor - Treatment category.
